# Supplementary material for: Modeling glioblastoma heterogeneity as a dynamic network of cell states
Source: Mol Syst Biol. 2021 Sep 16;17(9):e10105. doi: 10.15252/msb.202010105 (PMC8444284; doi:10.15252/msb.202010105)
Supplement: Supplementary file 6 — Source Data for Figure 5 [file MSB-17-e10105-s004.zip › Figure5A_sourcedata/GSEA_3017/nosigdb_stateB.GseaPreranked.1622795021725/RICHARDS_NOWAKOWSKI_SCIENCE_2017_MGE_IPC2_UPREG.html]

Details for gene set RICHARDS\_NOWAKOWSKI\_SCIENCE\_2017\_MGE\_IPC2\_UPREG[GSEA]

|  || Dataset | state43017 |
| Phenotype | NoPhenotypeAvailable |
| Upregulated in class | na\_pos |
| GeneSet | RICHARDS\_NOWAKOWSKI\_SCIENCE\_2017\_MGE\_IPC2\_UPREG |
| Enrichment Score (ES) | 0.56622046 |
| Normalized Enrichment Score (NES) | 3.6261141 |
| Nominal p-value | 0.0 |
| FDR q-value | 0.0 |
| FWER p-Value | 0.0 |
Table: GSEA Results Summary

  

Fig 1: Enrichment plot: RICHARDS\_NOWAKOWSKI\_SCIENCE\_2017\_MGE\_IPC2\_UPREG      
 Profile of the Running ES Score & Positions of GeneSet Members on the Rank Ordered List

  

| PROBE | GENE SYMBOL | GENE\_TITLE | RANK IN GENE LIST | RANK METRIC SCORE | RUNNING ES | CORE ENRICHMENT || 1 | HIST1H4C |  |  | 0 | 1.373 | 0.0118 | Yes |
| 2 | HIST1H1B |  |  | 1 | 1.272 | 0.0228 | Yes |
| 3 | HIST1H1D |  |  | 2 | 1.180 | 0.0330 | Yes |
| 4 | TOP2A |  |  | 3 | 1.079 | 0.0423 | Yes |
| 5 | HIST1H1C |  |  | 4 | 1.041 | 0.0513 | Yes |
| 6 | HIST1H1E |  |  | 5 | 0.983 | 0.0598 | Yes |
| 7 | RRM2 |  |  | 7 | 0.875 | 0.0654 | Yes |
| 8 | FAM111A |  |  | 10 | 0.813 | 0.0685 | Yes |
| 9 | UBE2C |  |  | 11 | 0.802 | 0.0754 | Yes |
| 10 | TPX2 |  |  | 12 | 0.801 | 0.0823 | Yes |
| 11 | NUSAP1 |  |  | 13 | 0.801 | 0.0892 | Yes |
| 12 | HMGB2 |  |  | 14 | 0.786 | 0.0960 | Yes |
| 13 | FBXO5 |  |  | 15 | 0.785 | 0.1028 | Yes |
| 14 | NCAPG |  |  | 16 | 0.778 | 0.1095 | Yes |
| 15 | SPC25 |  |  | 17 | 0.763 | 0.1161 | Yes |
| 16 | PRC1 |  |  | 18 | 0.756 | 0.1226 | Yes |
| 17 | CDCA2 |  |  | 19 | 0.751 | 0.1291 | Yes |
| 18 | ESCO2 |  |  | 20 | 0.742 | 0.1355 | Yes |
| 19 | GTSE1 |  |  | 22 | 0.732 | 0.1398 | Yes |
| 20 | HJURP |  |  | 23 | 0.731 | 0.1461 | Yes |
| 21 | KIF23 |  |  | 24 | 0.721 | 0.1523 | Yes |
| 22 | ASPM |  |  | 26 | 0.695 | 0.1564 | Yes |
| 23 | NDC80 |  |  | 27 | 0.695 | 0.1624 | Yes |
| 24 | ATAD2 |  |  | 28 | 0.685 | 0.1683 | Yes |
| 25 | KIF15 |  |  | 29 | 0.684 | 0.1742 | Yes |
| 26 | ARHGAP11A |  |  | 30 | 0.683 | 0.1801 | Yes |
| 27 | KPNA2 |  |  | 33 | 0.676 | 0.1820 | Yes |
| 28 | CENPF |  |  | 34 | 0.670 | 0.1878 | Yes |
| 29 | KIF4A |  |  | 37 | 0.662 | 0.1895 | Yes |
| 30 | ANLN |  |  | 39 | 0.659 | 0.1933 | Yes |
| 31 | RAD51AP1 |  |  | 40 | 0.657 | 0.1989 | Yes |
| 32 | SMC4 |  |  | 41 | 0.655 | 0.2046 | Yes |
| 33 | MELK |  |  | 42 | 0.655 | 0.2102 | Yes |
| 34 | CENPE |  |  | 43 | 0.654 | 0.2159 | Yes |
| 35 | NUF2 |  |  | 44 | 0.651 | 0.2215 | Yes |
| 36 | PRR11 |  |  | 45 | 0.649 | 0.2271 | Yes |
| 37 | DLGAP5 |  |  | 46 | 0.647 | 0.2327 | Yes |
| 38 | KIFC1 |  |  | 47 | 0.645 | 0.2382 | Yes |
| 39 | KIF18A |  |  | 48 | 0.639 | 0.2438 | Yes |
| 40 | DHFR |  |  | 49 | 0.638 | 0.2493 | Yes |
| 41 | NCAPG2 |  |  | 50 | 0.636 | 0.2548 | Yes |
| 42 | CDCA5 |  |  | 52 | 0.632 | 0.2582 | Yes |
| 43 | CKAP2 |  |  | 53 | 0.628 | 0.2637 | Yes |
| 44 | CCNA2 |  |  | 54 | 0.626 | 0.2691 | Yes |
| 45 | FANCI |  |  | 56 | 0.615 | 0.2724 | Yes |
| 46 | MYBL2 |  |  | 58 | 0.612 | 0.2757 | Yes |
| 47 | CKAP2L |  |  | 59 | 0.611 | 0.2810 | Yes |
| 48 | CENPU |  |  | 60 | 0.605 | 0.2862 | Yes |
| 49 | CENPK |  |  | 62 | 0.603 | 0.2895 | Yes |
| 50 | TYMS |  |  | 63 | 0.601 | 0.2946 | Yes |
| 51 | BUB1B |  |  | 64 | 0.596 | 0.2998 | Yes |
| 52 | KIF2C |  |  | 66 | 0.591 | 0.3029 | Yes |
| 53 | SMC2 |  |  | 68 | 0.586 | 0.3060 | Yes |
| 54 | CDK1 |  |  | 69 | 0.585 | 0.3111 | Yes |
| 55 | CKS1B |  |  | 72 | 0.575 | 0.3121 | Yes |
| 56 | RACGAP1 |  |  | 75 | 0.572 | 0.3131 | Yes |
| 57 | FOXM1 |  |  | 76 | 0.570 | 0.3180 | Yes |
| 58 | MAD2L1 |  |  | 77 | 0.569 | 0.3229 | Yes |
| 59 | DBF4 |  |  | 79 | 0.567 | 0.3258 | Yes |
| 60 | CDCA8 |  |  | 82 | 0.562 | 0.3267 | Yes |
| 61 | CKS2 |  |  | 83 | 0.558 | 0.3316 | Yes |
| 62 | ECT2 |  |  | 84 | 0.558 | 0.3364 | Yes |
| 63 | KIF14 |  |  | 85 | 0.557 | 0.3412 | Yes |
| 64 | E2F7 |  |  | 86 | 0.556 | 0.3460 | Yes |
| 65 | AURKB |  |  | 89 | 0.548 | 0.3468 | Yes |
| 66 | SPC24 |  |  | 91 | 0.544 | 0.3495 | Yes |
| 67 | CENPM |  |  | 92 | 0.544 | 0.3542 | Yes |
| 68 | CCNB1 |  |  | 93 | 0.544 | 0.3589 | Yes |
| 69 | NCAPH |  |  | 94 | 0.543 | 0.3636 | Yes |
| 70 | PBK |  |  | 95 | 0.540 | 0.3683 | Yes |
| 71 | DEPDC1 |  |  | 97 | 0.536 | 0.3709 | Yes |
| 72 | XRCC2 |  |  | 98 | 0.530 | 0.3755 | Yes |
| 73 | SKA3 |  |  | 99 | 0.525 | 0.3800 | Yes |
| 74 | TMEM106C |  |  | 100 | 0.523 | 0.3845 | Yes |
| 75 | HIST1H3D |  |  | 102 | 0.520 | 0.3871 | Yes |
| 76 | RRM1 |  |  | 103 | 0.520 | 0.3915 | Yes |
| 77 | CCNF |  |  | 106 | 0.513 | 0.3920 | Yes |
| 78 | CDKN3 |  |  | 108 | 0.513 | 0.3945 | Yes |
| 79 | CEP135 |  |  | 109 | 0.513 | 0.3989 | Yes |
| 80 | UBE2T |  |  | 110 | 0.512 | 0.4033 | Yes |
| 81 | CDCA3 |  |  | 111 | 0.511 | 0.4077 | Yes |
| 82 | DIAPH3 |  |  | 112 | 0.510 | 0.4121 | Yes |
| 83 | BUB1 |  |  | 113 | 0.508 | 0.4165 | Yes |
| 84 | TUBB4B |  |  | 114 | 0.507 | 0.4209 | Yes |
| 85 | RAD21 |  |  | 115 | 0.506 | 0.4253 | Yes |
| 86 | BIRC5 |  |  | 116 | 0.504 | 0.4296 | Yes |
| 87 | CLSPN |  |  | 117 | 0.503 | 0.4340 | Yes |
| 88 | DTYMK |  |  | 118 | 0.503 | 0.4383 | Yes |
| 89 | CEP152 |  |  | 119 | 0.502 | 0.4426 | Yes |
| 90 | KIF18B |  |  | 123 | 0.494 | 0.4410 | Yes |
| 91 | DUT |  |  | 124 | 0.494 | 0.4453 | Yes |
| 92 | E2F8 |  |  | 125 | 0.493 | 0.4495 | Yes |
| 93 | TMPO |  |  | 127 | 0.492 | 0.4518 | Yes |
| 94 | ITGB3BP |  |  | 128 | 0.490 | 0.4560 | Yes |
| 95 | SKA1 |  |  | 131 | 0.487 | 0.4563 | Yes |
| 96 | GMNN |  |  | 135 | 0.484 | 0.4546 | Yes |
| 97 | NCAPD2 |  |  | 136 | 0.483 | 0.4587 | Yes |
| 98 | TTK |  |  | 137 | 0.482 | 0.4629 | Yes |
| 99 | FANCB |  |  | 138 | 0.478 | 0.4670 | Yes |
| 100 | CENPL |  |  | 139 | 0.478 | 0.4711 | Yes |
| 101 | C21orf58 |  |  | 142 | 0.470 | 0.4713 | Yes |
| 102 | FANCD2 |  |  | 149 | 0.461 | 0.4635 | Yes |
| 103 | ORC6 |  |  | 150 | 0.461 | 0.4674 | Yes |
| 104 | KIF22 |  |  | 152 | 0.459 | 0.4694 | Yes |
| 105 | NCAPD3 |  |  | 153 | 0.458 | 0.4734 | Yes |
| 106 | SPAG5 |  |  | 156 | 0.455 | 0.4734 | Yes |
| 107 | MCM8 |  |  | 158 | 0.454 | 0.4753 | Yes |
| 108 | GPC6 |  |  | 159 | 0.454 | 0.4793 | Yes |
| 109 | POLQ |  |  | 162 | 0.450 | 0.4792 | Yes |
| 110 | CENPQ |  |  | 163 | 0.446 | 0.4831 | Yes |
| 111 | TK1 |  |  | 167 | 0.444 | 0.4810 | Yes |
| 112 | BRIP1 |  |  | 168 | 0.443 | 0.4848 | Yes |
| 113 | ATAD5 |  |  | 170 | 0.442 | 0.4867 | Yes |
| 114 | BRCA1 |  |  | 171 | 0.440 | 0.4905 | Yes |
| 115 | CDK5RAP2 |  |  | 173 | 0.438 | 0.4923 | Yes |
| 116 | BRCA2 |  |  | 174 | 0.436 | 0.4960 | Yes |
| 117 | SMCHD1 |  |  | 175 | 0.436 | 0.4998 | Yes |
| 118 | RBBP8 |  |  | 176 | 0.436 | 0.5036 | Yes |
| 119 | DEPDC1B |  |  | 177 | 0.436 | 0.5073 | Yes |
| 120 | NCAM1 |  |  | 178 | 0.436 | 0.5111 | Yes |
| 121 | MKI67 |  |  | 179 | 0.436 | 0.5149 | Yes |
| 122 | FEN1 |  |  | 180 | 0.435 | 0.5186 | Yes |
| 123 | CDC20 |  |  | 184 | 0.431 | 0.5164 | Yes |
| 124 | USP1 |  |  | 185 | 0.430 | 0.5201 | Yes |
| 125 | MIS18BP1 |  |  | 186 | 0.430 | 0.5238 | Yes |
| 126 | MGME1 |  |  | 188 | 0.428 | 0.5256 | Yes |
| 127 | BARD1 |  |  | 189 | 0.427 | 0.5293 | Yes |
| 128 | CKAP5 |  |  | 192 | 0.426 | 0.5290 | Yes |
| 129 | MND1 |  |  | 193 | 0.424 | 0.5327 | Yes |
| 130 | NEK2 |  |  | 194 | 0.423 | 0.5363 | Yes |
| 131 | MIS18A |  |  | 196 | 0.421 | 0.5380 | Yes |
| 132 | CCDC15 |  |  | 197 | 0.419 | 0.5416 | Yes |
| 133 | MCM4 |  |  | 199 | 0.418 | 0.5432 | Yes |
| 134 | DSN1 |  |  | 202 | 0.413 | 0.5429 | Yes |
| 135 | SYNE2 |  |  | 203 | 0.413 | 0.5464 | Yes |
| 136 | ZWINT |  |  | 212 | 0.407 | 0.5342 | Yes |
| 137 | LIG1 |  |  | 214 | 0.407 | 0.5358 | Yes |
| 138 | CCNE2 |  |  | 215 | 0.406 | 0.5393 | Yes |
| 139 | H2AFZ |  |  | 216 | 0.405 | 0.5428 | Yes |
| 140 | UTRN |  |  | 220 | 0.400 | 0.5403 | Yes |
| 141 | CENPO |  |  | 223 | 0.398 | 0.5398 | Yes |
| 142 | TIMELESS |  |  | 228 | 0.395 | 0.5354 | Yes |
| 143 | STIL |  |  | 231 | 0.393 | 0.5348 | Yes |
| 144 | PCM1 |  |  | 232 | 0.393 | 0.5382 | Yes |
| 145 | EZH2 |  |  | 236 | 0.388 | 0.5357 | Yes |
| 146 | VRK1 |  |  | 238 | 0.388 | 0.5371 | Yes |
| 147 | PARPBP |  |  | 240 | 0.387 | 0.5384 | Yes |
| 148 | PCNA |  |  | 242 | 0.386 | 0.5398 | Yes |
| 149 | HMGN2 |  |  | 243 | 0.386 | 0.5431 | Yes |
| 150 | POLA1 |  |  | 247 | 0.383 | 0.5406 | Yes |
| 151 | RTKN2 |  |  | 248 | 0.379 | 0.5438 | Yes |
| 152 | CCNB2 |  |  | 250 | 0.379 | 0.5451 | Yes |
| 153 | JADE1 |  |  | 251 | 0.378 | 0.5484 | Yes |
| 154 | POLD3 |  |  | 252 | 0.377 | 0.5516 | Yes |
| 155 | MPHOSPH9 |  |  | 254 | 0.375 | 0.5529 | Yes |
| 156 | PLK4 |  |  | 255 | 0.374 | 0.5561 | Yes |
| 157 | RAD18 |  |  | 256 | 0.373 | 0.5594 | Yes |
| 158 | C18orf54 |  |  | 259 | 0.371 | 0.5586 | Yes |
| 159 | CENPJ |  |  | 261 | 0.370 | 0.5599 | Yes |
| 160 | GAS2L3 |  |  | 262 | 0.369 | 0.5630 | Yes |
| 161 | TUBB |  |  | 263 | 0.368 | 0.5662 | Yes |
| 162 | CENPW |  |  | 267 | 0.366 | 0.5635 | No |
| 163 | SMC1A |  |  | 274 | 0.361 | 0.5548 | No |
| 164 | CHAF1A |  |  | 276 | 0.359 | 0.5559 | No |
| 165 | MMS22L |  |  | 277 | 0.359 | 0.5590 | No |
| 166 | ANP32E |  |  | 280 | 0.358 | 0.5582 | No |
| 167 | PSIP1 |  |  | 284 | 0.357 | 0.5554 | No |
| 168 | HAUS8 |  |  | 293 | 0.353 | 0.5427 | No |
| 169 | RFC4 |  |  | 295 | 0.351 | 0.5438 | No |
| 170 | DTL |  |  | 305 | 0.345 | 0.5291 | No |
| 171 | CDC25C |  |  | 307 | 0.343 | 0.5301 | No |
| 172 | CENPN |  |  | 309 | 0.343 | 0.5311 | No |
| 173 | FAM111B |  |  | 314 | 0.342 | 0.5262 | No |
| 174 | DEK |  |  | 316 | 0.341 | 0.5271 | No |
| 175 | RFC3 |  |  | 322 | 0.338 | 0.5202 | No |
| 176 | HAUS6 |  |  | 324 | 0.337 | 0.5212 | No |
| 177 | CIT |  |  | 326 | 0.336 | 0.5221 | No |
| 178 | RFC2 |  |  | 331 | 0.333 | 0.5171 | No |
| 179 | HIRIP3 |  |  | 336 | 0.331 | 0.5121 | No |
| 180 | BTG3 |  |  | 337 | 0.331 | 0.5150 | No |
| 181 | UBR7 |  |  | 340 | 0.330 | 0.5139 | No |
| 182 | HAT1 |  |  | 352 | 0.324 | 0.4951 | No |
| 183 | TMEM237 |  |  | 353 | 0.324 | 0.4979 | No |
| 184 | ARHGAP11B |  |  | 356 | 0.323 | 0.4967 | No |
| 185 | LMNB1 |  |  | 363 | 0.321 | 0.4877 | No |
| 186 | XPO1 |  |  | 365 | 0.321 | 0.4885 | No |
| 187 | TOPBP1 |  |  | 369 | 0.319 | 0.4854 | No |
| 188 | NCAPH2 |  |  | 377 | 0.315 | 0.4743 | No |
| 189 | PRIM2 |  |  | 391 | 0.311 | 0.4515 | No |
| 190 | KIF20A |  |  | 392 | 0.311 | 0.4542 | No |
| 191 | TMEM245 |  |  | 394 | 0.310 | 0.4549 | No |
| 192 | CEP192 |  |  | 395 | 0.310 | 0.4576 | No |
| 193 | BLM |  |  | 408 | 0.305 | 0.4366 | No |
| 194 | CENPC |  |  | 409 | 0.304 | 0.4392 | No |
| 195 | LIN54 |  |  | 411 | 0.303 | 0.4399 | No |
| 196 | PTTG1 |  |  | 413 | 0.302 | 0.4405 | No |
| 197 | ZGRF1 |  |  | 415 | 0.302 | 0.4412 | No |
| 198 | AHI1 |  |  | 425 | 0.299 | 0.4261 | No |
| 199 | NUP107 |  |  | 430 | 0.298 | 0.4208 | No |
| 200 | G2E3 |  |  | 446 | 0.294 | 0.3938 | No |
| 201 | IQGAP3 |  |  | 447 | 0.294 | 0.3964 | No |
| 202 | CCDC150 |  |  | 449 | 0.293 | 0.3969 | No |
| 203 | CBX5 |  |  | 457 | 0.291 | 0.3857 | No |
| 204 | SAE1 |  |  | 463 | 0.289 | 0.3784 | No |
| 205 | RFWD3 |  |  | 464 | 0.289 | 0.3809 | No |
| 206 | NUP155 |  |  | 468 | 0.288 | 0.3775 | No |
| 207 | KCTD9 |  |  | 471 | 0.287 | 0.3760 | No |
| 208 | YEATS4 |  |  | 476 | 0.286 | 0.3706 | No |
| 209 | CHEK1 |  |  | 477 | 0.286 | 0.3731 | No |
| 210 | RNASEH2A |  |  | 478 | 0.285 | 0.3755 | No |
| 211 | NUCKS1 |  |  | 483 | 0.285 | 0.3701 | No |
| 212 | HADH |  |  | 485 | 0.284 | 0.3706 | No |
| 213 | APOLD1 |  |  | 487 | 0.284 | 0.3711 | No |
| 214 | KCNJ2 |  |  | 490 | 0.283 | 0.3696 | No |
| 215 | HMGXB4 |  |  | 491 | 0.282 | 0.3721 | No |
| 216 | POLH |  |  | 492 | 0.282 | 0.3745 | No |
| 217 | CTDSPL2 |  |  | 495 | 0.282 | 0.3730 | No |
| 218 | PSMC3 |  |  | 506 | 0.280 | 0.3558 | No |
| 219 | WDHD1 |  |  | 513 | 0.278 | 0.3464 | No |
| 220 | TTF2 |  |  | 514 | 0.278 | 0.3488 | No |
| 221 | CDC27 |  |  | 520 | 0.278 | 0.3413 | No |
| 222 | LSM3 |  |  | 522 | 0.277 | 0.3418 | No |
| 223 | DNMT1 |  |  | 523 | 0.277 | 0.3442 | No |
| 224 | SMARCA2 |  |  | 530 | 0.276 | 0.3348 | No |
| 225 | POLR2A |  |  | 545 | 0.273 | 0.3096 | No |
| 226 | ALYREF |  |  | 546 | 0.273 | 0.3120 | No |
| 227 | SAP30 |  |  | 547 | 0.273 | 0.3143 | No |
| 228 | SCLT1 |  |  | 548 | 0.273 | 0.3167 | No |
| 229 | ACYP1 |  |  | 551 | 0.273 | 0.3151 | No |
| 230 | CDH2 |  |  | 558 | 0.271 | 0.3056 | No |
| 231 | BRD8 |  |  | 562 | 0.270 | 0.3021 | No |
| 232 | CDK2 |  |  | 565 | 0.270 | 0.3005 | No |
| 233 | NUP205 |  |  | 571 | 0.269 | 0.2930 | No |
| 234 | CNTRL |  |  | 575 | 0.269 | 0.2894 | No |
| 235 | ST8SIA5 |  |  | 588 | 0.266 | 0.2681 | No |
| 236 | IDH2 |  |  | 598 | 0.263 | 0.2527 | No |
| 237 | CEP57 |  |  | 605 | 0.262 | 0.2432 | No |
| 238 | ZNF519 |  |  | 618 | 0.260 | 0.2219 | No |
| 239 | ARL6IP6 |  |  | 629 | 0.258 | 0.2044 | No |
| 240 | CENPI |  |  | 630 | 0.258 | 0.2067 | No |
| 241 | EXOSC8 |  |  | 647 | 0.256 | 0.1774 | No |
| 242 | UHRF1 |  |  | 652 | 0.255 | 0.1718 | No |
| 243 | RDX |  |  | 658 | 0.255 | 0.1642 | No |
| 244 | PCBP2 |  |  | 660 | 0.255 | 0.1644 | No |
| 245 | SRBD1 |  |  | 661 | 0.254 | 0.1666 | No |
| 246 | FAM13B |  |  | 674 | 0.252 | 0.1452 | No |
| 247 | SPATA5 |  |  | 675 | 0.252 | 0.1474 | No |
| 248 | MYBL1 |  |  | 678 | 0.251 | 0.1456 | No |
| 249 | CHD7 |  |  | 706 | -0.284 | 0.0950 | No |
| 250 | DSEL |  |  | 709 | -0.297 | 0.0936 | No |
| 251 | SOX4 |  |  | 712 | -0.305 | 0.0923 | No |
Table: GSEA details [plain text format]

  

Fig 2: RICHARDS\_NOWAKOWSKI\_SCIENCE\_2017\_MGE\_IPC2\_UPREG: Random ES distribution      
 Gene set null distribution of ES for **RICHARDS\_NOWAKOWSKI\_SCIENCE\_2017\_MGE\_IPC2\_UPREG**
